# The six-question Gastroesophageal Reflux Disease Questionnaire (GerdQ) cannot accurately quantify reflux and reflux-associated symptoms frequency

**DOI:** 10.1093/gastro/goac043

**Published:** 2022-08-19

**Authors:** Thomas J Hurr

**Affiliations:** South Australian Reflux Research Unit, Adelaide, South Australia, Australia

The aim of this commentary is to demonstrate, using two examples, that the six-question Gastroesophageal Reflux Disease Questionnaire (GerdQ) cannot accurately quantify reflux and reflux-associated symptoms (RRAS) frequency [1].

A systematic review identified 65 distinct questionnaires for the assessment of gastroesophageal reflux disease (GERD) including the GerdQ [2]. The GerdQ has become a widely used questionnaire to diagnose GERD and quantify reflux symptom frequency and treatment response (Supplementary Table 1) [1].

The GerdQ has six questions (6GerdQ) with a Likert scale for positive predictors (Questions 1, 2, 5, and 6) being on a scale of 0–3 and a reversed Likert scale for negative predictors (Questions 3 and 4) being on a scale of 3–0 giving a total GerdQ score from 0 to 18 with a recommended cut-off score of ≥8 to diagnose GERD [1].

The two positive predictors summarized as Question 1, heartburn, and Question 2, regurgitation, are considered the two characteristic reflux symptoms of GERD and form the two-question GerdQ (2GerdQ). The reflux-associated symptoms, Question 5, sleep disturbance, and Question 6, over-the-counter medication use in addition to that prescribed, are considered a measure of disease impact and when combined with Questions 1 and 2 form the four-question GerdQ (4GerdQ). The 4GerdQ is considered to measure RRAS.

The two negative predictors of GERD summarized as Question 3, pain in the centre of the upper stomach, and Question 4, nausea, are not considered RRAS but are used to diagnose GERD by their absence. The absence of symptoms for Questions 3 and 4 result in a score of 6 and this is used to predict a diagnosis of GERD. The presence of symptoms for Questions 3 and 4 can give a score of 0, suggesting that these symptoms are not characteristic or negatively predict a diagnosis of GERD. As Questions 3 and 4 are not considered RRAS and scored on a reverse Likert scale, the 6GerdQ total score cannot then be considered to quantify RRAS frequency.

To demonstrate that the 6GerdQ total scores cannot accurately quantify RRAS frequency, based on possible scoring patterns and a recommended cut-off score of ≥8, an example is shown (Table 1). Patient A has a positive predictor score of 5 (Q1 + Q2) and a negative predictor score of 6 (Q3 + Q4) with the negative predictors indicating no symptoms over the previous week giving an overall score of 11.

**Table 1.** An example of possible patient scores using the 6GerdQ to diagnose gastroesophageal reflux disease (GERD) with a cut-off score of ≥8. Patient A is diagnosed with GERD (score of 11) and patient B is not (score of 6) despite patient B having more reflux symptoms than patient A due to a higher positive predictor score.

Questions from the 6GerdQ	Patient A with GERD	Patient B without GERD
Q1 positive predictor	3	3
Q2 positive predictor	2	3
Q3 negative predictor	3	0
Q4 negative predictor	3	0
Q5 positive predictors	0	0
Q6 positive predictor	0	0
Total score	11	6

Patient B has a positive predictor and overall score of 6 (Q1 + Q2) and therefore patient B has a higher RRAS frequency than patient A, as only the positive predicting scores indicate RRAS frequency. The 6GerdQ predicts patient A has GERD and patient B does not, for a cut-off score of ≥8, despite patient A having a lower score for RRAS frequency. This is because patient B has a score of 0 for the negative predictors (Q3 + Q4) with pain in the centre of the upper stomach and nausea 4–7 days a week—symptoms that negatively predict GERD (Table 1). The example from Table 1 shows that negative predictors should not be used to quantify RRAS frequency because they can increase the total 6GerdQ score, without the score reflecting an increase in RRAS. The recommended cut-off score of ≥8 required for a diagnosis of GERD from the 6GerdQ is too high for patient B, who has high scores for the two characteristic reflux symptoms (Q1 + Q2 = 6) and significant reflux symptoms, but fails to be diagnosed with GERD.

The 4GerdQ can evaluate RRAS frequency or a treatment response based on a change in RRAS score with a score of 0 representing no RRAS and a score of 12 representing the highest RRAS frequency [3]. For the 4GerdQ, a new cut-off score has been defined as sufficient relief by a score of ≤1 and complete resolution by a score of 0 [3].

An example of the benefit of using only the 4GerdQ and the 2GerdQ rather than the 6GerdQ is shown in Figure 1 using the data from an *N*-of-1 trial evaluating treatment response [4]. The trial compared treatment A, 80 mg esomeprazole (40 mg mornings or AM and 40 mg evenings or PM), with treatment B (40 mg AM and placebo PM) over 12 weeks [4]. For simplicity, only the original data from the 6GerdQ scores for treatment A are compared with the 4GerdQ and 2GerdQ (Figure 1). Using the 4GerdQ and the 2GerdQ, the patient is shown to have a greater response to treatment and is asymptomatic during Week 5. The asymptomatic patient score of 6 from the 6GerdQ is not a suitable end point, which would typically be expected to be 0, to show complete symptom resolution and a successful treatment response. The difference between the 4GerdQ and the 2GerdQ is considered a measure of reflux disease impact (Figure 1) [1].

**Figure 1.**
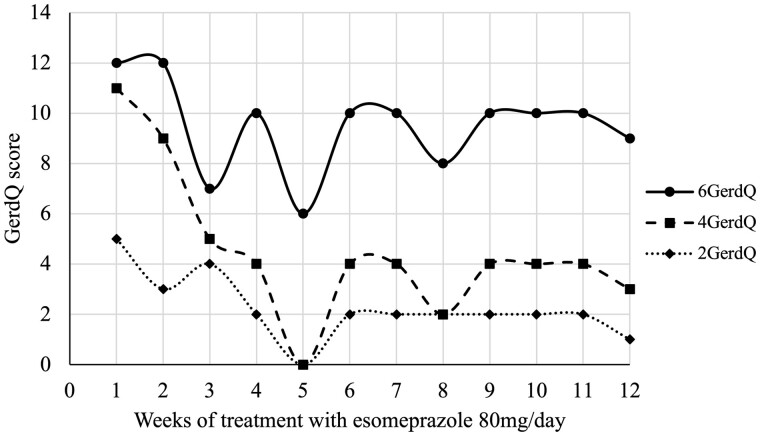
Results for a 12-week, *N*-of-1 trial of treatment A, esomeprazole (40 mg AM and 40 mg PM) using the 6GerdQ. Scores from the 4GerdQ and the 2GerdQ are shown for comparison. Note the patient is asymptomatic during Week 5 [4]. The 6GerdQ is the sum of the frequency scores from Questions 1–6; 4GerdQ, the sum of the frequency scores from Questions 1, 2, 5, and 6; 2GerdQ, the sum of the frequency scores from Questions 1 and 2.

The original authors, over a decade ago, reported that both the 6GerdQ and 4GerdQ could be used to diagnose GERD and accurately measure treatment response for GERD over time, but for the 6GerdQ this is not the same as quantifying RRAS frequency [1]. Since the original publication, many authors have used the 6GerdQ values to quantify RRAS frequency and assumed that a change in this score represents a change in RRAS frequency. As Questions 3 and 4 are not considered symptoms of reflux and so are scored on a reverse Likert scale, it has been shown, from the examples, that this can result in an increase in the 6GerdQ score, without representing an increase in RRAS frequency.

In conclusion, as the total scores from the 6GerdQ failed to diagnose patient B (Table 1) as having GERD based on the recommended cut-off score of ≥8, despite having significant reflux symptoms, it may be time to consider whether the use of the 6GerdQ should be discontinued in favour of the 4GerdQ and 2GerdQ to diagnose GERD and quantify RRAS.

## Supplementary Data

Supplementary data is available at *Gastroenterology Report* online.

## Funding

The author has not received funding for this work.

## Conflict of Interest

None declared.

## Supplementary Material

goac043_Supplementary_Data
