# Supplementary material for: The six-question Gastroesophageal Reflux Disease Questionnaire (GerdQ) cannot accurately quantify reflux and reflux-associated symptoms frequency
Source: Gastroenterol Rep (Oxf). 2022 Aug 19;10:goac043. doi: 10.1093/gastro/goac043 (PMC9390063; doi:10.1093/gastro/goac043)
Supplement: goac043_Supplementary_Data [file goac043_supplementary_data.docx]

**Supplementary Table 1.** The 6 question GerdQ (6GerdQ) where respondents enter the frequency scores after reflecting on their symptoms over the previous week [1]. Republished with permission of John Wiley & Sons from *Aliment Pharmacol Ther*. Development of the GerdQ, a tool for the diagnosis and management of gastro-oesophageal reflux disease in primary care. 2009;30:1030-1038, through Copyright Clearance Center Inc. Table 1 is not covered by the terms of the Creative Commons licence of this publication. For permission reuse, please contact the rightsholder.

| Question | Frequency score (points) for symptoms | | | |
| --- | --- | --- | --- | --- |
|  | 0 day | 1 day | 2–3 days | 4–7 days |
| 1. How often did you have a burning feeling behind your breastbone (heartburn)? | 0 | 1 | 2 | 3 |
| 2. How often did you have stomach contents (liquid or food) moving upwards to your throat or mouth (regurgitation)? | 0 | 1 | 2 | 3 |
| 3. How often did you have a pain in the centre of the upper stomach? | 3 | 2 | 1 | 0 |
| 4. How often did you have nausea? | 3 | 2 | 1 | 0 |
| 5. How often did you have difficulty getting a good night’s sleep because of your heartburn and/or regurgitation? | 0 | 1 | 2 | 3 |
| 6. How often did you take additional medication for your heartburn and/or regurgitation, other than what the physician told you to take? | 0 | 1 | 2 | 3 |
